# A Low Red/Far-Red Light Ratio Promotes a Reduction in Time from Sowing to Flowering in Wheat Under Speed Breeding Conditions

**DOI:** 10.3390/plants14233614

**Published:** 2025-11-26

**Authors:** Valeriya M. Nagamova, Daria O. Bizyakina, Andrey O. Blinkov, Yana V. Minkova, Nataliya Yu. Svistunova, Svetlana Radzeniece, Aleksey S. Yanovsky, Alina A. Kocheshkova, Mikhail G. Divashuk

**Affiliations:** 1 All-Russia Research Institute of Agricultural Biotechnology, 127550 Moscow, Russia; vnagamova01@mail.ru (V.M.N.); dasha.biz@mail.ru (D.O.B.); flleurs@mail.ru (Y.V.M.); aloevera3006@gmail.com (N.Y.S.); radzen.lana@gmail.com (S.R.); alina.korotaeva@gmail.com (A.A.K.); divashuk@gmail.com (M.G.D.); 2 P.P. Lukyanenko National Grain Centre, Central Estate of KNIISH, 350012 Krasnodar, Russia; yanovskij81@list.ru

**Keywords:** durum wheat, far-red light, photomorphogenesis, red light, speed breeding

## Abstract

Conventional methods for obtaining pure durum wheat lines are time-consuming and low-throughput, making speed breeding (SB) a promising alternative. This study investigated SB optimization using far-red (FR) light. Plants were grown under three red/far-red (R/FR) ratios (6.6, 1.0, 0.4) and on three substrates (peat, soil mixture, mineral wool). Reducing the R/FR ratio significantly accelerated flowering, with the most substantial reduction (R/FR = 0.4) shortening the time to flowering by 4.1–4.2 days. The extent of this acceleration and a concurrent negative impact on spike productivity (vegetative weight of dried spikes, the number of spikelets, and the number of grains per spike) were both dependent on the substrate type. Furthermore, a positive correlation was found between the duration of the sowing-to-flowering period and spike productivity components (spike length and number of grains per spike). Increasing the proportion of FR light enhanced the 1000-grain weight and did not affect the germination rate or regenerative capacity. Modifying the SB for durum wheat by adding FR light (R/FR = 0.4) is a useful strategy for increasing its efficiency, and the negative impact of FR light can be mitigated by adjusting mineral nutrition.

## 1. Introduction

One of the limiting factors in durum wheat breeding is the time required to develop pure lines. The most common approach for obtaining homozygous breeding lines (F_5_–F_6_) of this crop is pedigree method, which can take up to 9–15 years from the initial hybridization to testing of the developed varieties in various agroecological zones [1]. Moreover, this issue is also relevant in genetic research on durum wheat, such as the development of recombinant inbred lines (RILs, F_6_) to identify and study quantitative trait loci (QTL) [2,3], and in the creation of near-isogenic lines (NILs) to study the effects of genes [4,5].

Researchers have always sought approaches to reduce the time required to develop pure lines. One of the most effective methods to solve this problem is doubled haploid technology [6]. This method proved ineffective for durum wheat and is associated with a low frequency of haploid embryo formation and a high rate of albino plant regeneration [1,7,8,9]. Durum wheat shuttle breeding increases selection efficiency but does not substantially reduce the time required to develop pure lines [10].

The most recent breakthrough approach for rapidly obtaining homozygous plant forms is speed breeding. This method is based on the application of factors that reduce the time from sowing to flowering (photoperiod, light sources, spectral composition, light intensity, temperature, carbon dioxide, vernalization, mineral nutrition, substrate volume, mechanical tillering removal, plant growth regulators), shorten the duration of the generative stage of development, and overcome post-harvest seed dormancy (embryo culture, the germination of immature seeds, or the artificial drying of seeds with subsequent heating, scarification, stratification, or gibberellic acid treatment) [11,12,13]. This method has also proven effective for durum wheat, enabling the production of up to six generations per year [12,14,15,16].

Currently, active work is underway to modify speed breeding protocols to enhance their efficiency [13,17,18,19,20,21]. However, most speed breeding protocols avoid the use of far-red light, despite its potency as an inducer of a significant reduction in the vegetative period.

The ratio of red (R) to far-red (FR) light (660 nm and 730 nm, respectively) differentially influences plant growth and development. Light radiation at these wavelengths is perceived by the family of phytochrome photoreceptors (*Phy*A, *Phy*B and *Phy*C), which are involved in the photoperiodic regulation of flowering. Daylight contains approximately equal proportions of red and far-red light (R/FR = 1.0–1.3). Under leaf and forest canopies, the ratio of red to far-red light decreases sharply. This decrease is associated with the active absorption of red light by photosynthetic pigments and the reflection of far-red light from leaves. In this case, a low R/FR ratio serves as an indicator of the proximity of competing neighbors and triggers the shade avoidance syndrome. This syndrome manifests as enhanced elongation growth, reorientation of leaves towards regions of unattenuated daylight, and accelerated flowering induction, all of which increase plant survival [22,23,24].

Experiments in plant physiology have shown that the targeted application of supplemental FR light can also induce shade avoidance syndrome, leading to the time reduction from sowing to flowering and decrease tillering shoot growth [25,26,27]. It can also reduce the number of fertile flowers and grain number per spike [21,25,28], as well as promote internode elongation [29,30]. The extent of change in the listed growth parameters depends not only on the presence of far-red light but also on its ratio to red light. The greatest influence on the changes in plant growth and development parameters is exerted by the ratio in which R:FR < 1 [23,28,31]. On the other hand, the application of FR light in combination with shorter wavelengths (400–680 nm) can enhance photosynthetic efficiency and, consequently, plant productivity, a phenomenon described as the Emerson effect [32,33,34].

Furthermore, far-red light affects plant mineral nutrition [23]. The application of this light reduces root hair density and root biomass [35,36,37]. Far-red light can also inhibit mycorrhiza formation, which affects the uptake of nitrogen and phosphorus [38]. The reduced nitrogen assimilation under a high proportion of far-red light is also explained by the effect of this light on the enzymes nitrate reductase, nitrite reductase, and glutamine synthetase [23,39].

The efficacy of FR light application in speed breeding has been demonstrated for crops such as rapeseed [40], amaranth [29], and pepper [30]. Depending on the crop and the proportion of far-red light, the reduction in the vegetative period can range from 3 [29] to 56 [40] days. However, the influence of FR light on cereals under speed breeding conditions has not been investigated. Published speed breeding protocols for cereals exhibit a range of FR light incorporation, from its complete absence [12,41] to its presence in low [11,42] or high proportions [43]. Furthermore, almost no comprehensive studies have been conducted to investigate the influence of far-red light on the physiology of durum wheat [44,45,46].

Therefore, the objectives of this study are to evaluate the effects of far-red light, at different ratios to red light, on the duration of the vegetative period and the productivity of durum wheat under speed breeding conditions in various types of substrates.

## 2. Results

### 2.1. The Effect of Far-Red Light on the Vegetative Period of Durum Wheat

**Figure 1:**
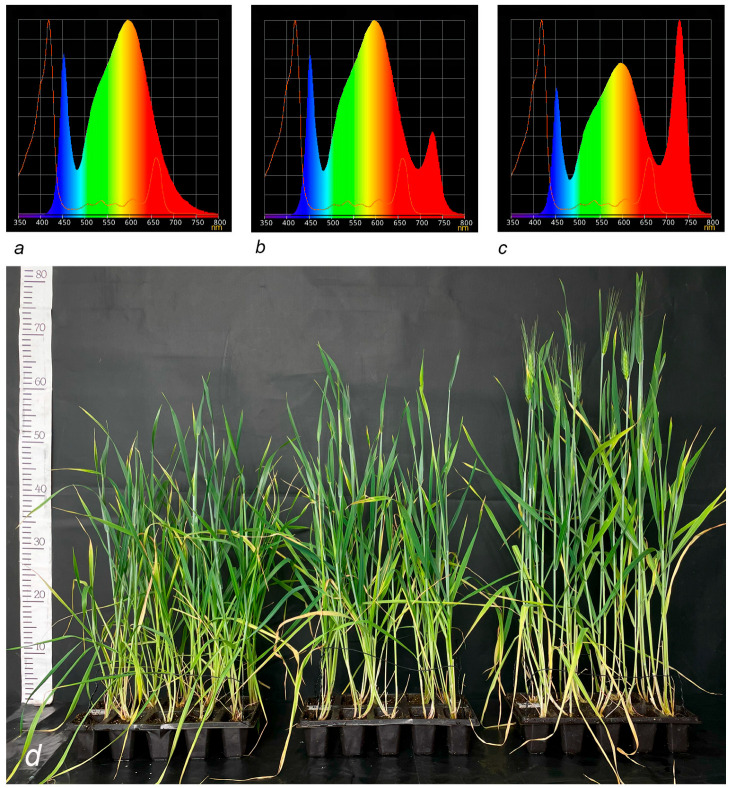
Spectral composition of light used in the experiment: (**a**)—R > FR, R/FR ratio = 6.6; (**b**)—R = FR, R/FR ratio = 1.0; (**c**)—R < FR, R/FR ratio = 0.4; (**d**)—Plants of the durum wheat variety Yasenka, sown on the same day and grown under lighting with different spectral compositions: R/FR = 6.6—plants on the left; R/FR = 1.0—plants in the center; R/FR = 0.4—plants on the right.

The results of the two-way ANOVA test showed statistically significant differences at the 5% significance level in the heading and flowering dates for different spectral compositions and substrate types. The experiment revealed a statistically significant (*p* < 0.05) reduction in the duration of the vegetative period across all substrate types for plants grown under the spectral composition with the highest proportion of far-red light (R/FR = 0.4) compared to the spectrum with the lowest proportion (R/FR = 6.6) (Table 1). Specifically, in plants exposed to the spectrum with the highest far-red light proportion (R/FR = 0.4), heading occurred 4.0 to 7.1 days earlier and flowering occurred 4.1 to 4.2 days earlier, depending on the substrate used, compared to the spectrum with the minimal far-red light proportion (R/FR = 6.6) (Figure 1d). A similar trend of a reduction in the time from sowing to flowering was observed in plants grown under the R/FR = 1.0 spectral composition. Under these conditions, plants grown in peat and soil mixture flowered statistically significantly earlier, by approximately 2.6 to 3.6 days (*p* < 0.05), than those under the R/FR = 6.6 spectrum (Table 1). No significant differences (*p* > 0.05) in the timing of flowering were found for durum wheat plants grown on mineral wool under the R/FR = 1.0 and R/FR = 6.6 lighting regimes. Under all lighting regimens, there was a statistically significant difference in the time from sowing to flowering between substrates: plants in mineral wool flowered significantly later than those in peat or soil mixture.

According to four-year cultivation data for the Krasnodar region, the durum wheat variety Yasenka reached heading 84.0 ± 10.0 days after sowing.

### 2.2. The Effect of Far-Red Light on Yield Components of Durum Wheat

The results of the two-way ANOVA showed statistically significant differences in the vegetative weight of the dried spike, 1000-grain weight, number of grains per spike (*p* < 0.001), and number of spikelets per spike (*p* < 0.01) among different spectral compositions and substrate types. According to the two-way ANOVA, no statistically significant difference in spike length was found depending on light conditions and substrate type (*p* > 0.05).

A negative effect of an increased proportion of far-red light on a number of parameters related to the plant reproductive system was detected. When mineral wool was used, plants grown under R/FR = 0.4 exhibited a statistically significant reduction in the vegetative mass of spikes and the number of grains per spike compared to plants grown under R/FR = 6.6. In plants grown on peat, a decrease in the number of spikelets and grains per spike was observed under the light spectrum with the highest proportion of far-red light (R/FR = 0.4) compared to those under the spectrum with R/FR = 6.6. When a soil mixture was used, only the number of spikelets per spike was reduced under the R/FR = 0.4 light treatment compared to the R/FR = 6.6 treatment.

A statistically significant positive effect of lighting with a high proportion of far-red light (R/FR = 0.4) on the 1000-grain weight was observed in plants grown on the soil mixture and mineral wool.

No statistically significant differences were observed in plant productivity parameters for plants grown on different substrates under the light spectra with R/FR = 6.6 and R/FR = 1.0, except for a decrease in the number of spikelets per spike in plants grown on peat and an increase in the 1000-grain weight in plants grown on the soil mixture under the R/FR = 1.0 spectrum (Table 2).

**Figure 2:**
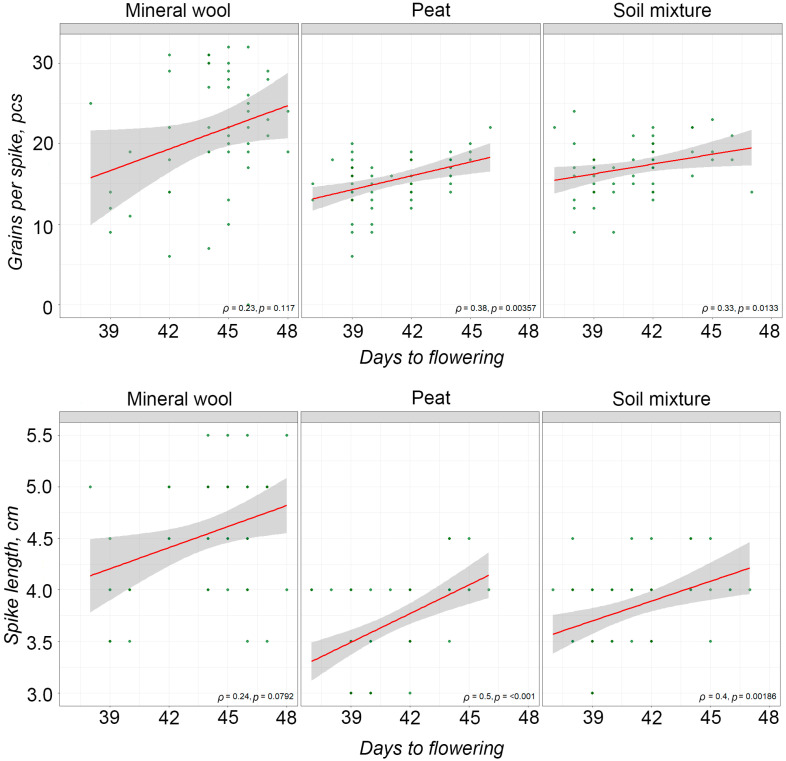
Correlation plots between the duration of the time from sowing to flowering and spike productivity components. Green dots—original values of the observed parameters for each individual plant; the red line—trend line (an upward slope indicates a positive correlation); the gray area—95% confidence interval.

A statistically significant positive correlation was found between the duration of the time from sowing to flowering and spike productivity components (spike length and number of grains per spike). According to Spearman’s correlation, a significant, moderately strong positive relationship was observed between the duration of the time from sowing to flowering and spike length in plants grown on peat and the soil mixture (ρ = 0.5, *p* < 0.001 and ρ = 0.4, *p* < 0.01, respectively). A similar relationship was found between the duration of the time from sowing to flowering and the number of grains per spike (ρ = 0.38, *p* < 0.01 and ρ = 0.33, *p* < 0.05, respectively) (Figure 2).

### 2.3. The Effect of Far-Red Light on Seed Viability and Germination

Two-way ANOVA showed no statistically significant differences in the regenerative capacity of isolated embryos obtained from plants grown on different substrates and under different spectral compositions (*p* > 0.05). Across all lighting treatments of the donor plants, embryos with coleoptiles and roots were observed as early as the third day of cultivation. By the tenth day of cultivation, the embryos had developed one fully formed leaf and a well-developed root system. The frequency of embryo regeneration into viable plants was 100 ± 0% across all lighting treatments and substrates used (Table S1).

Also, no statistically significant effect of far-red light on seed germination was found in seeds obtained from durum wheat plants grown using different substrates (*p* > 0.05). Mean germination rates ranged from 93.0 ± 6.7% to 100 ± 0%. All germinated seeds exhibited well-formed roots and coleoptiles (Table S1).

## 3. Discussion

The results of our experiment demonstrate that far-red light in the optical radiation spectrum has a statistically significant influence on reduction of the vegetative period of durum wheat. Durum wheat flowered significantly earlier when the highest proportion of far-red light was used (R/FR = 0.4). The data indicate that under speed breeding conditions, a higher proportion of far-red light leads to a greater reduction in the time from sowing to heading and flowering. Therefore, for a significant reduction in the time from sowing to flowering in durum wheat under the long-day conditions of speed breeding, not only is the presence of far-red light important, but also its proportion when it substantially exceeds that of red light. A similar trend of a reduced vegetative period with an increased proportion of far-red light has also been reported in a number of other studies [26,47,48].

Our work revealed an interaction between the substrate and the light spectrum on the sowing-to-flowering duration: plants grown on mineral wool flowered statistically significantly later than those on peat or soil mixture. The data we obtained suggest that in speed breeding, it is possible to modify the substrate composition or mineral nutrition to enhance the impact of a high far-red light proportion on the length of the vegetative period.

The sowing-to-flowering period for durum wheat was significantly shorter under the spectrally modified protocol (R/FR = 0.4), with plants reaching heading 47.5–48.1 days earlier than those grown under the field conditions of the Krasnodar region.

Although the difference in flowering time in our study was 4.1–4.2 days, far-red light can be considered a valuable additional factor in establishing speed breeding conditions for durum wheat. If the time from sowing to flowering is reduced by four days in a single generation, the cumulative effect over the sequential cultivation of six generations—typically sufficient to obtain a pure line [49,50,51]—could reach 24 days. It is important to note that this is a scenario-based extrapolation, contingent on the consistent realization of this reduction in each generation and not accounting for operational constraints that would be encountered in a practical breeding pipeline. Therefore, this value should be viewed as a theoretical maximum under idealized conditions.

Our results are consistent with those of other studies showing the efficacy of far-red light application for reducing the vegetative period under speed breeding conditions. Currently, the effectiveness of far-red light for modifying speed breeding protocols has been demonstrated only for dicot crops, such as rapeseed [40], amaranth [29], and pepper [30].

Our analysis revealed a statistically significant negative impact of a high proportion of far-red light on the spike productivity components of durum wheat. Plants grown under an increased proportion of far-red light (R/FR = 0.4) exhibited a tendency for reduced vegetative weight, and the decrease in the number of spikelets and grains per spike. Similar results have been reported in bread wheat, where an increased amount of far-red light reduces the number of fertile flowers and grains per spike [25,28]. The extent of the reduction in spike productivity parameters due to the high proportion of far-red light also depended on the type of substrate in which the plants were grown. When a soil mixture was used, only a statistically significant decrease in the number of spikelets per spike was observed in plants grown under a high proportion of far-red light. This finding indicates that the substrate composition, which directly affects plant mineral nutrition, can mitigate the negative effect of far-red light on spike productivity parameters. The negative influence of far-red light on plant nutrition has been previously demonstrated in a number of crops [23,35,39,52].

Subsequent analysis enabled us to identify a significant, moderately strong positive correlation between the duration of time from sowing to flowering and spike productivity components (spike length and number of grains per spike). A shorter vegetative period in plants was associated with shorter spikes and fewer grains per spike (Figure 2). A decrease in plant yield under speed breeding conditions has also been previously reported [12]. A yield reduction associated with a shorter vegetative period under field conditions has been described for numerous agricultural crops, including durum wheat [53,54]. Based on the obtained data, it can be suggested that the observed reduction in productivity may be associated with the shorter vegetative period under speed breeding conditions.

The negative impact of an increased far-red light proportion on the reproductive system of durum wheat cannot be considered a limiting factor for its use in the spectral composition of light in speed breeding. This is because the one of the most common breeding schemes used in speed breeding systems for developing pure lines is single-seed descent, which requires only one seed per plant to proceed to the next breeding cycle [12,13,14].

Despite the negative impact of far-red light on spike productivity components, plants grown under an increased proportion of far-red light in the optical radiation spectrum exhibited a significantly higher 1000-grain weight. This could be attributed to Emerson effect, which involves an enhancement in photosynthetic efficiency when far-red light is used in combination with shorter wavelengths (400–680 nm) [32]. Supplementing the optical radiation spectrum with far-red light leads to an increase in certain elements of productivity in a number of crops [32,33,34]. This, in turn, can be a distinct advantage of using far-red light in speed breeding, beyond just reducing the time from sowing to flowering. Forming under high far-red light proportions (R/FR = 0.4), the wheat seeds did not differ in the regenerative properties of the embryos or in germination. Thus, using a light spectrum with an increased proportion of far-red light will not limit the transition to the next breeding cycle.

It should be taken into account that the reduction in time from sowing to flowering of 4.1–4.2 days, accompanied by simultaneous changes in spike characteristics when using a spectral composition with R/FR = 0.4, is directly related to the cultivar and growing conditions. The use of other durum wheat genotypes, as well as changes in cultivation parameters, will lead to alterations in both the duration of the sowing-to-flowering period and the characteristics associated with spike productivity.

To date, limited publications are available on the influence of far-red light on durum wheat. This study demonstrated the effect of far-red light on the rate of cell division during leaf formation, tillering, and plant height [44,45,46]. Our study provides additional data that contribute not only to the potential modification of the speed breeding protocol for durum wheat but also to the understanding of far-red light’s influence on the physiology of this crop.

Data obtained on the influence of far-red light on the vegetative period of durum wheat under speed breeding conditions could be useful for modifying speed breeding protocols for other long-day cereals.

## 4. Materials and Methods

### 4.1. Plant Material and Growth Conditions

The spring durum wheat (*Triticum durum* Desf.) variety Yasenka, developed by Lukyanenko National Grain Centre (Krasnodar, Russia), was selected for this study.

Prior to sowing, all the seeds were treated with the fungicide Maxim (Syngenta, Saint-Pierre-la-Garenne, France). The treated seeds were germinated on moist filter paper in the dark at +25 °C. After several days, the germinated seeds were transferred to one of three growing substrates:

Substrate 1: Peat “Agrobalt-S” (Pindstrup, Zaplyusye, Russia), (N 150 mg/L; P_2_O_5_ 150 mg/L; K_2_O 250 mg/L; Ca 120 mg/L; Mg 30 mg/L; pH—5), (40 g of moistened peat per tray cell).

Substrate 2: Soil mixture consisting of peat (N 150 mg/L; P_2_O_5_ 150 mg/L; K_2_O 250 mg/L; Ca 120 mg/L; Mg 30 mg/L; pH—5), chernozem (N 27.8 mg/kg; P_2_O_5_ 94 mg/kg; K_2_O 123 mg/kg; 6–7% humus; pH—6.5–7.5), sand, and vermiculite in a 5:3:1:1 ratio (50 g of moistened mixture per tray cell).

Substrate 3: Mineral wool cubes measuring 50 × 45 × 45 mm (one cube per tray cell).

The planting was conducted using 10-cell trays with a volume of 110 mL per cell, placing a single seed in each cell at a substrate depth of 1 cm. One seed was placed in each cell of the tray.

The plants were grown in an accelerated-growth room (Gorshkoff, Moscow, Russia). Throughout the vegetative period, a photoperiod of 22/2 h day/night was maintained. The temperature was kept constant at +24 ± 1 °C, and the relative air humidity at 40 ± 5%.

During the first two weeks of cultivation, plants grown in Substrates 1 and 2 were watered daily as needed. Root fertilization with Tripart fertilizer (Terra Aquatica, Fleurance, France) for Substrates 1 and 2 was applied weekly according to the manufacturer’s instructions. From the beginning of the third week, fertilization for Substrates 1 and 2 was increased to three times per week. Plants grown on Substrate 3 were irrigated with this fertilizer solution daily from the moment of seedling emergence. Weekly foliar feeding with Siliplant (Nest-M, Moscow, Russia) was applied to plants in Substrates 1, 2, and 3, following the manufacturer’s instructions. Treatments against diseases and pests were applied as necessary.

### 4.2. The Influence of Far-Red Light on Plant Growth Period Duration and Productivity

The extent of far-red light’s influence on durum wheat was determined by cultivating plants under three lighting regimes provided by LED lamps (Prometheus, Zheleznodorozhny, Russia). The lamps were positioned 85 cm above the table where the plants were placed. The regimes differed in the ratio of radiation levels in the 660 nm region (R—red) and 730 nm (FR—far-red):

1. R/FR ratio = 6.6 (hereinafter referred to as R > FR) (Control) (Figure 1a; Table S2);
2. R/FR ratio = 1.0 (hereinafter referred to as R = FR) (Figure 1b; Table S2);
3. R/FR ratio = 0.4 (hereinafter referred to as R < FR) (Figure 1c; Table S2).

The light intensity for all treatments was constant, set to a PPFD of ~330 μmol/(m^2^·s) at the center of the shelf where the plants were placed, throughout the 22-h photoperiod. Far-red light was switched on one week after seed germination. The lighting parameters were calibrated and verified using PG200N spectrometer (United Power Research Technology Corp., Zhunan, Taiwan). Before each use of the spectrometer, a dark calibration was performed according to the manufacturer’s instructions. Measurements of light intensity and spectral composition were carried out at three time points: prior to arranging the plant trays, immediately after turning on the far-red light, and then repeated weekly after far-red light exposure.

The onset of phenological phases was assessed for each plant individually according to [55]. The onset of the heading stage was defined as the day when the spike fully emerged from the flag leaf sheath (stage Z5.9). The onset of the flowering stage was defined as the day when anthers first became visible on the spikes (stage Z6.1). The experiment was conducted with 20 plants for each substrate and for each spectral composition. To analyze the effect of light spectral composition on the development rate of durum wheat, the number of days from sowing to heading and from sowing to flowering was recorded for each individual plant.

To evaluate the influence of far-red light and substrate type on durum wheat, yield components were assessed using the following parameters: spike length (cm), vegetative weight of the dried spike (g), number of spikelets (pcs.) and grains (pcs.) per spike, and weight of 1000 grains (g).

### 4.3. Plant Growth Under Field Conditions

Data on heading (stage Z5.9) from long-term field trials (2020–2023) at the P.P. Lukyanenko National Grain Center (Krasnodar region, Russia) were used as an additional control. Agronomic practices and sowing dates were conventional for the region.

### 4.4. The Effect of Far-Red Light on Seed Viability

The influence of far-red light on seed viability parameters was assessed using two methods: (1) by culturing immature embryos in vitro and (2) by germinating seeds on filter paper.

In the first method, embryo isolation was performed 15 days after flowering. Caryopses were surface-sterilized for 20 min in a 50% solution of the commercial agent “Belizna”, followed by three rinses in sterile distilled water. All manipulations with embryos were carried out using an Olympus SZ61 stereoscopic microscope (Olympus, Tokyo, Japan). The isolated embryos were placed in Petri dishes containing Murashige and Skoog (MS) agar medium [56] and sealed with Parafilm. Cultivation was conducted under a 22/2 h day/night photoperiod. A light intensity of ~80 μmol/(m^2^·s) was maintained at the level of the Petri dishes, which were placed on a shelf 28 cm from the light source. The temperature was kept constant at +24 °C. Regeneration was assessed on the 10th day after isolation. The experiment employed four replicates with 10 isolated embryos each.

In the second method, spikes were harvested 20 days after flowering and placed in paper bags. Prior to harvesting, irrigation was gradually phased out over a five-day period. The spikes in paper bags were force-dried in the temperature-controlled incubator TSO-1/80 (SKTB-SPU, Smolensk, Russia) at +28 °C for one week or longer, depending on the drying rate. After drying, the spikes were threshed, and the seeds were stored for three weeks at room temperature in paper bags. Seeds were placed in Petri dishes on moistened filter paper one month after drying and subjected to stratification at +4 °C for three days in the dark. A vernalization chamber (Klimbiotech, Moscow, Russia) was used for this process. Following the cold treatment, the Petri dishes were kept in the dark at +25 °C in the temperature-controlled incubator TSO-1/80 (SKTB-SPU, Russia). Germination capacity was assessed on the seventh day after transferring the dishes to +25 °C. Seed germination was evaluated with four replicates of 50 seeds each.

### 4.5. Statistical Analysis

The data were processed using the R programming language (version 4.3.2). The normality of the distribution was checked using the Shapiro–Wilk test. The effect of light spectral composition and substrate on various parameters of durum wheat was assessed using two-way Align Rank Transform (ART) ANOVA. When statistically significant differences were detected, a post hoc Dunn’s test was performed with a Bonferroni correction for seed germination rate and a Benjamini–Hochberg correction for vegetation rate and spike productivity parameters. To identify dependencies between the time from sowing to flowering and spike productivity components (spike length and number of grains per spike), Spearman’s rank correlation coefficient was calculated.

## 5. Conclusions

The results of this study demonstrate that the following:

(1) Not only the presence of far-red light but also its ratio to red light affects the shortening of the vegetative period in durum wheat. The spectral composition with the highest proportion of far-red light (R/FR~0.4) had the greatest effect on reducing the sowing-to-heading period.
(2) A negative impact of far-red light on durum wheat spike productivity parameters was revealed. A statistically significant positive correlation was found between the duration of the sowing-to-heading period and both spike length and the number of grains per spike.
(3) An interaction between the factors of light spectral composition and substrate on plant growth rate and productivity was shown. This relationship indicates that modifying mineral nutrition could potentially be used to either enhance the effect of far-red light on shortening the vegetative period or to mitigate its negative impact on spike productivity.
(4) A trend towards an increase in the 1000-grain weight was observed when a high proportion of far-red light (R/FR~0.4) was used.
(5) The absence of any effect of far-red light on the regenerative capacity of isolated embryos and seed germination was demonstrated.
(6) The use of a high proportion of far-red light (R/FR~0.4) could be an effective tool for modifying durum wheat speed breeding protocols, allowing the vegetative period to be shortened by 4.1–4.2 days.
(7) The obtained results reveal several promising directions for future research. Firstly, modifying the light spectrum by incorporating a high proportion of far-red light holds potential for optimizing speed breeding protocols, not only for cereals but also for other crops. Secondly, this approach could form the basis for developing such protocols for plant species where speed breeding methods are currently unavailable. Finally, the identified interaction between the substrate’s mineral composition and far-red light suggests the possibility of optimizing these protocols through nutritional management. This could enhance the positive effect of far-red light on shortening the vegetative phase while mitigating its potential negative impact on productivity.

## Figures and Tables

**Table 1:** The effects of spectral composition and substrate type on heading and flowering dates.

Days from Sowing to Heading				Days from Sowing to Flowering			
R/FR Ratio	Mineral Wool	Peat	Soil Mixture	R/FR Ratio	Mineral Wool	Peat	Soil Mixture
R/FR = 6.6	43.4 ± 1.0 ^1^ d ^2^	40.5 ± 0.9 c	41.4 ± 1.2 c	R/FR = 6.6	45.7 ± 1 d	43.6 ± 0.7 c	43.5 ± 1.0 c
R/FR = 1.0	41.4 ± 0.9 c	37.2 ± 0.7 ab	37.8 ± 0.7 b	R/FR = 1.0	44.6 ± 0.6 cd	40.0 ± 0.8 ab	40.9 ± 0.8 ab
R/FR = 0.4	36.3 ± 0.7 ab	36.5 ± 0.6 ab	35.9 ± 0.7 a	R/FR = 0.4	41.6 ± 1.3 b	39.4 ± 0.6 a	39.3 ± 0.7 a

^1^ Mean values ± 95% confidence interval for heading and flowering dates in plants of the durum wheat variety Yasenka. ^2^ Values followed by the same letter are not significantly different (*p* > 0.05) according to Dunn’s post-hoc test with Benjamini–Hochberg correction.

**Table 2:** The effects of spectral composition and substrate type on yield component parameters.

R/FR Ratio	Mineral Wool	Peat	Soil Mixture
Spike length, cm			
R/FR = 6.6	4.8 ± 0.3 ^ns^	4.1 ± 0.1	4.1 ± 0.1
R/FR = 1.0	4.7 ± 0.2	3.6 ± 0.1	3.9 ± 0.2
R/FR = 0.4	4.2 ± 0.2	3.4 ± 0.2	3.6 ± 0.2
Vegetative weight of the dried spike, g			
R/FR = 6.6	1.28 ± 0.12 ^1^ d ^2^	1.00 ± 0.07 bc	1.05 ± 0.07 bcd
R/FR = 1.0	1.12 ± 0.10 cd	1.01 ± 0.04 bc	1.08 ± 0.08 cd
R/FR = 0.4	0.71 ± 0.2 a	0.84 ± 0.08 ab	1.04 ± 0.16 bcd
1000-grain weight, g			
R/FR = 6.6	33.0 ± 2.3 a	40.9 ± 2.5 bc	39.6 ± 3.3 b
R/FR = 1.0	34.2 ± 1.9 a	44.8 ± 1.7 cd	46.6 ± 2.3 d
R/FR = 0.4	45.7 ± 1.5 cd	43.7 ± 2.7 bcd	45.1 ± 2.0 cd
Number of grains per spike, pcs.			
R/FR = 6.6	25.8 ± 2.5 e	17.3 ± 1.1 bc	18.5 ± 1.6 cd
R/FR = 1.0	22.5 ± 2.8 de	16.1 ± 0.9 abc	16.7 ± 1.7 abc
R/FR = 0.4	13.4 ± 4.3 ab	12.8 ± 1.5 a	16.3 ± 1.2 abc
Number of spikelets per spike, pcs			
R/FR = 6.6	11.5 ± 0.7 de	11.6 ± 0.5 de	11.2 ± 0.6 cde
R/FR = 1.0	11.8 ± 0.4 e	9.4 ± 0.7 ab	9.9 ± 0.8 abc
R/FR = 0.4	10.3 ± 0.6 bcd	8.6 ± 0.9 a	9.1 ± 0.8 ab

^ns^ Non-significant according to two-way ANOVA (*p* > 0.05). ^1^ Mean values ± 95% confidence interval for yield component parameters of the Yasenka variety. ^2^ Values followed by the same letter are not significantly different (*p* > 0.05) according to Dunn’s post-hoc test with Benjamini–Hochberg correction.

## Data Availability

The original contributions presented in this study are included in the article. Further inquiries can be directed to the corresponding author.

## Supplementary Materials

The following supporting information can be downloaded at: https://www.mdpi.com/article/10.3390/plants14233614/s1, Table S1: Seed germination rate and regenerative capacity of isolated embryos; Table S2: Spectral power distributions for all spectral compositions.

## Abbreviations

The following abbreviations are used in this manuscript:

R	Red light
FR	Far-red light
R/FR	Red-to-Far-red ratio
PPFD	Photosynthetic Photon Flux Density
